# Prevalence, Antibiotic Susceptibility Pattern and Associated Factors of *Streptococcus pyogenes* among Pediatric Patients with Acute Pharyngitis in Sidama, Southern Ethiopia

**DOI:** 10.1155/2024/9282571

**Published:** 2024-09-17

**Authors:** Alemitu Beyene Gebre, Demissie Assegu Fenta, Abel Abera Negash, Betelihem Jima Hayile

**Affiliations:** ^1^ Hawassa University College of Medicine and Health Science School of Medical Laboratory Science, Hawassa, Ethiopia; ^2^ Armauer Hansen Research Institute, Addis Ababa, Ethiopia

## Abstract

**Background:**

*Streptococcus pyogenes* is the most frequent cause of pharyngitis and skin infections in children and causes immune complications like rheumatic fever and rheumatoid heart disease (RHD), particularly in developing countries like Ethiopia. The aim of this study was to determine the prevalence, antibiotic resistance pattern, and associated factors of *Streptococcus pyogenes* among pediatric patients suspected of acute pharyngitis in Sidama Region, Southern Ethiopia.

**Methods:**

A cross-sectional study was conducted on 213 acute pharyngitis suspected pediatric patients from April to September 2022 at Hawassa University Compressive Specialized Hospital and Yirgalem Hospital. Sociodemographic and clinical data were collected using a structured questionnaire. A throat swab was cultured to isolate *S*. *pyogenes*, and antimicrobial susceptibility testing was done using standard bacteriological techniques. Data were analyzed using SPSS version 25, and *P* value of <0.05 was considered as statistically significant.

**Result:**

Out of 213 throat swabs cultured, 22 (10.3%) with 95% CI (6.6–14.6%) were *S*. *pyogenes* positive. All isolates of *S*. *pyogenes* were sensitive to penicillin and amoxicillin. In contrast, 8 (36.4%) isolates exhibited resistance to tetracycline, 7 (31.8%) to ceftriaxone, 6 (27.3%) to erythromycin, and 5 (22.7%) isolates showed multidrug resistance. The presence of palatal petechiae (*P*=0.037) and tonsillar swelling or exudate (*P*=0.007) were significantly associated with *S*. *pyogenes* carriage in children suspected of having acute pharyngitis.

**Conclusion:**

In this study, the prevalence of *S*. *pyogenes* among children suspected with acute pharyngitis was low compared to other studies. The isolates showed a high level of resistance to commonly used antibiotics. Therefore, the treatment of pediatric acute *S*. *pyogenes* pharyngitis should depend on an antimicrobial susceptibility test. Furthermore, evaluation of *S*. *pyogenes* pediatric acute pharyngitis risk factors and tracking of antibiotic resistance are crucial in the controlling of pediatric acute *S*. *pyogenes* pharyngitis.

## 1. Introduction

*Streptococcus pyogenes* (*S*. *pyogenes*) is a Gram-positive, facultative anaerobic, *β*-hemolytic bacterium classified as group A streptococcus based on carbohydrate it contains [1, 2]. It is the most frequent cause of skin and oropharyngeal infections, which can develop into more serious invasive and suppurative conditions like toxic shock syndrome and necrotizing fasciitis, which have been associated to increased rates of morbidity and mortality globally [3–5]. Of these infections, pharyngitis is the most common among children, particularly those between the ages of 5 and 14 years. It causes 288.6 million episodes, with a high episode rate of 40 per 100 child in the low- and middle-income countries (LMICs), translating into 0.1 million disability-adjusted living years (DALYs) yearly [6]. The frequency of *S*. *pyogenes* pharyngitis was highest in sub-Saharan Africa, with a rate of 5.7 per 1000, as compared to 1.8 per 1000 in North Africa and 0.3 per 1000 in developed nations [7]. For example, a 2017 study conducted in Uganda found that of 600 million cases of pharyngitis, 450 million cases were thought to be caused by *S*. *pyogenes* per year, with 20 to 40% of those cases being in children [8], despite, there are few publications of *S*. *pyogenes* in Africa [9].

*S*. *pyogenes* is a contagious organism and can spread through direct or indirect contact with respiratory tract secretions, wounds, or sores on infected people's skin [10]. The infection varies depending on a number of factors, including age, gender, and standard of living in addition to seasonal variations and indoor air pollution [11]. *S*. *pyogenes* remains susceptible to penicillin, and penicillin has proven to be an effective empirical treatment antibiotic for *S*. *pyogenes* infection. However, patients who have developed allergies to penicillin are treated with alternative antibiotics such as cephalosporin, lincosamides, and macrolides drugs [12, 13]. However, currently there have been varying reports of *S*. *pyogenes* resistance to cephalosporin, lincosamides, and macrolides (erythromycin and azithromycin) [4, 12, 14]. This antibiotic resistance of *S*. *pyogenes* is mainly associated with drug overuses and poststreptococcus infection complication such as glomerulonephritis, rheumatic fever (RF), and rheumatic heart disease (RHD) which is the main concern worldwide [15, 16].

In Ethiopia, data regarding *S*. *pyogenes* in children suspected with pharyngitis and antibiotic resistance are extremely limited, especially in the study area. The earlier study conducted in the study area found that 12% of children had asymptomatic pharyngeal carriage of *S*. *pyogenes* [17]. This was somewhat higher than the 9.1–11.3% prevalence of *S*. *pyogenes* among children in other regions who were suspected of having pharyngitis [17–19] and demand great attention. Additionally, both nationally and in the study area, empirical medications are the mainstay of treatment for *S*. *pyogenes* pharyngitis in Ethiopia. Despite *S*. *pyogenes* pharyngitis infection's high resistance to clindamycin, ceftriaxone, cefotaxime, cefepime, and erythromycin, antibiotics are prevalent in Ethiopia [18]. Also, tetracycline and azithromycin resistance was highly observed in both asymptomatic and symptomatic *S*. *pyogenes* pharyngitis infections [17, 19, 20] in addition to multidrug resistant (MDR) cases [18]. Therefore, the aim of this study was to determine the prevalence of *S*. *pyogenes*, antibiotic susceptibility pattern, and associated factors among children suspected with acute pharyngitis in Sidama, Southern Ethiopia.

## 2. Materials and Methods

### 2.1. Study Area, Design, and Period

A hospital-based cross-sectional study was conducted at two government hospitals: Hawassa University Comprehensive Specialized Hospital (HUCSH) and Yirgalem General Hospital, in Sidama region, Southern Ethiopia, from April 15 to September 10, 2022. Hawassa University Comprehensive Specialized Hospital (HUCSH) is located at Hawassa City which is 275 km from Addis Ababa, the capital city of Ethiopia. It is the only comprehensive specialized hospital in Southern Ethiopia and provides different health services for more than 18 million peoples in Sidama region, Southern Nations, Nationalities, and Peoples (SNNPR), Southern Oromia, and some part of Somalia regions. Similarly, Yirgalem General Hospital which is located 322 km from Addis Ababa and 47 km from Hawassa City, the second largest hospital in Sidama region gives different health services in the catchment area and the community from surrounding.

### 2.2. Study Population

All pediatric patients who were attending pediatric emergency wards in both hospitals during the study period were the study population.

### 2.3. Inclusion and Exclusion Criteria

All children under 18 years of age with symptoms of acute pharyngitis whose parents accepted the consent were included in this study; however, children who have been on antibiotics in the last two weeks were excluded from the study [18].

### 2.4. Sample Size Determination and Sampling Techniques

Sample size was determined using the following assumptions: 95% confidence interval (CI), power of 80%, and previous odds ratio of associated factors from Bahir Dar [18], by using EPI INFO stat calc version 7 software. To minimize errors arising from the likelihood of noncompliance, 10% of the sample size was added. Finally, by considering the 10% nonresponse rate, the sample size calculated was 213. A systematic random sampling technique was applied to select 213 respondents for interview.

### 2.5. Sampling Procedure and Sampling Technique

The participant's proportional allocation was calculated based on previous child flow data due to acute pharyngitis per month from each hospital's logbook. A systematic random sampling technique was applied to select 213 children who were suspected of having acute pharyngitis: 142 from HUCSH and 71 from Yirgalem General Hospital. To choose the first participant, the K interval was calculated. After the K interval was calculated, the first individual was selected by the lottery method and the others at a regular interval.

### 2.6. Operational Definition

Multidrug resistance (MDR): a species of microorganisms that is resistant to at least one agent in ≥3 antimicrobial categories [21].

Acute pharyngitis: a clinical feature characterized by sudden onset of sore throat, palatal petechiae, anterior cervical lymphadenopathy, and tonsillar swelling with or without exudates [22].

### 2.7. Collection of Sociodemographic and Clinical Data

Sociodemographic characteristics data from the parents or children, as well as the children's clinical history, were collected by trained nurses using a structured questionnaire under physician supervision during children acute pharyngitis clinical examination.

### 2.8. Sample Collection and Transportation Methods

Two throat samples were collected by trained nurses, one for Gram staining and the other for culture purpose, by using a sterile cotton swab and a sterile disposable tongue depressor from each child under physician supervision. Each collected swab was immediately immersed in the Amies transport medium (Oxoid, England). Samples were then transported to the Hawassa University School of Medical Laboratory, Microbiology laboratory within half an hour at room temperature from the Hawassa University Comprehensive Specialized Hospital, and using a cold bag from Yirgalem General Hospital.

### 2.9. *Streptococcus pyogenes* Identification

The throat swab was inoculated on blood agar plates (blood agar base Oxoid UK) by rolling the swab over a small area of the plate and streaking the sample using a sterile loop. The plates were then incubated for 24 hours at 37°C in a candle jar, which can provide an atmosphere of 5% CO_2_. A plate culture that was negative for beta-hemolytic colonies after 24 hours was incubated for an additional 24 hours to allow the growth of slow growers. Beta-hemolytic streptococci were checked by their colony morphology, beta-hemolysis, and gram stain. Gram-positive and catalase-negative streptococcus bacteria were confirmed by bacitracin disc sensitivity and pyrolidonyl arylamidase (PYR) tests. Bacitracin-sensitive and a purple color in PYR tests were identified as *S. pyogenes* [23].

### 2.10. Antimicrobial Susceptibility Testing

An antimicrobial susceptibility test was performed by the disc diffusion method using Mueller–Hinton agar (MHA) (Oxoid UK) supplemented with 5% sheep blood according to the Clinical Laboratory Standards Institute (CLSI) 2021 guidelines [24]. Colony suspension was made using normal saline (0.85% NaCl) equivalent to 0.5% McFarland standard from colonies grown for 24 hrs on a sheep blood agar plate. The suspension was inoculated on the MHA plate with 5% sheep blood using a culture swab and incubated at 5% CO_2_ for 24 hours. Based on the CLSI 2021 guideline, ceftriaxone (30 *μ*g), cefepime (30 *μ*g), clindamycin (2 *μ*g), erythromycin (15 *μ*g), penicillin (10 IU), amoxicillin (10 *µ*g), chloramphenicol (30 *µ*g), and tetracycline (30 *μ*g) antibiotic-impregnated disks (Abteck Biologicals Ltd) were selected. Finally, the zone of inhibition was measured with a ruler and interpreted according to the CLSI 2021 guideline value.

### 2.11. Statistical Analysis

Data were entered into Epi Info version 3.1 and analyzed using SPSS version 25. *S*. *pyogenes* prevalence, variable frequency, and antimicrobial susceptibility pattern were determined by descriptive statistics. Bivariate logistic regression analysis was used to determine the associated risk factors. For those variables which have a *P* value <0.25 in the bivariate, the analysis was further entered into the multivariable logistic regression model and a *P* value of <0.05 was considered statistically significant at 95% CI.

### 2.12. Data Quality Control

Prior to actual data collection, the quality of the data was assured by pretesting 5% of questionnaires at Adare General Hospital, Hawassa City. Training was given to the data collector's physician and nurses. For the laboratory test, the sterility of prepared culture media was checked by incubating the 5% of prepared media in a 5% CO_2_-enriched atmosphere at 37°C for 24 hours. For each test, a quality control strain of *S. pyogenes* (ATCC19615) from the Ethiopian Public Health Institute (EPHI) was used as the positive control to check all media, biochemical tests, and antibiotics. To maintain the quality of the work and data management, the standard operating procedure for sample collection and laboratory analysis was strictly followed.

### 2.13. Ethical Approval and Consent to Participate

Ethical approval was obtained from the Institutional Review Board of Hawassa University, College of Medicine and Health Sciences (Ref. No: IRB/010/14). Permission was obtained from the clinical and academic director of HUCSH and Yirgalem General Hospital. Before enrolling, any of the eligible study participants' written informed consent was obtained from the children's parents or caregivers, and assent was also obtained from minors. Finally, all procedures were conducted based on the ethical Declaration of Helsinki guidelines, and all information was kept confidential. All positive results were reported to the Pediatrics Emergency Department of HUCSH and Yirgalem General Hospital physicians for appropriate antibiotic treatment.

## 3. Results

### 3.1. Sociodemographic Characteristics of Study Participants

This study included 213 children who were suspected of having acute pharyngitis. Among the children included in this study, 114 (53.1%) were males. The mean age of the study participants was 5.7, with a standard deviation (SD) of ±3.22 years. Of the participants, 199 (93.4%) were from urban areas. Regarding clinical characteristics, out of the 213 children, 138 (64.8%), 17 (8.0%), and 25 (11.7%) had a history of tonsillitis, otitis media, and conjunctive, respectively. Moreover, 138 (64.8%) of them had petechiae on the palate, 83 (39.0%) had enlarged anterior cervical lymph nodes, and 90 (42.3%) had tonsillar swelling or exudate (Table 1).

The overall prevalence of *S*. *pyogenes* was 22 (10.3%) and was more predominant in males, 17 (8.0%) than females. *S*. *pyogenes* acute pharyngitis was 12 (5.6%) more prevalent in children aged <5 years. Among the children with culture proven *S*. *pyogenes* pharyngitis, 15 (7.0%) of their parents were working in private occupations. Furthermore, *S*. *pyogenes* pharyngitis was more prevalent among children with a previous history of tonsil, 15 (7.0%), and tonsillar swelling or exudate, 14 (6.6) (Table 1).

### 3.2. Antimicrobial Susceptibility Testing Patterns

**Figure 1:**
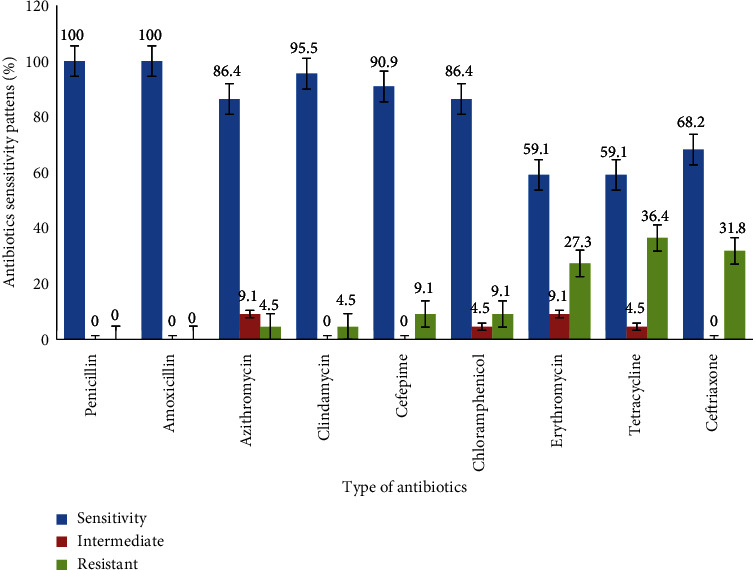
Antibiotic susceptibility pattern of *S*. *pyogenes* among pediatric patients with acute pharyngitis at HUCSH and Yirgalem General Hospital, 2022.

All *S*. *pyogenes* isolates were sensitive to both penicillin and amoxicillin. The highest rate of drug resistance was observed for tetracycline, 36.4% (8/22) followed by ceftriaxone, 31.8% (7/22), and erythromycin, 27.3% (6/22) (Figure 1).

Overall, in this study, 5/22 (22.7%) of the *S*. *pyogenes* isolates showed multidrug resistance (MDR). Of those, 1 (4.5%) exhibited resistance to erythromycin, ceftriaxone, and tetracycline and about 1 (4.5%) was resistant to clindamycin, azithromycin, and tetracycline, while the other 1 (4.5%) demonstrated resistance to ceftriaxone, chloramphenicol, erythromycin, and tetracycline. Additionally, the left two individuals (9.1%) exhibited resistance to erythromycin, ceftriaxone, cefepime, and tetracycline (Table 2).

### 3.3. Factors Associated with *S*. *pyogenes* Pharyngitis

In bivariate analysis, sex, age, parent occupation, number of windows, tonsillar swelling or exudate, nasal flow, and vomiting were found to be statistically significant at a *P* value of <0.25 and were therefore taken into consideration as potential candidates for multivariate analysis. After adjusting other confounding variables, boys' children were 3.91 times more likely to develop *S*. *pyogenes* acute pharyngitis than girls' children (AOR = 3.91; 95% CI = 1.297–11.792; *P*=0.015), children with palatal petechiae were 65% more likely to develop *S*. *pyogenes* acute pharyngitis than without (AOR = 0.35; 95% CI = 0.131–0.938; *P*=0.037), and children with tonsillar swelling or exudate were 3.83 times more likely to develop *S*. *pyogenes* acute pharyngitis than those who have not developed tonsillar swelling or exudate (AOR = 3.83; 95% CI = 1.446–10.188, *P*=0.007) (Tables 3 and 4).

## 4. Discussion

*S*. *pyogenes*, the most common cause of acute pharyngitis infections, causes an annual incidence of over 600 million and is associated with morbidity and mortality worldwide [4]. In this study, the overall prevalence of S. pyogenes was 22 (10.3%), with 95% CI (6.6–14.2%). This finding was comparable with the Ethiopian previous studies' result of different areas, Jimma, 11.3% [19], Gondar, 10.7% [20], Bahir Dar, 9.1% [18], and another country, Indonesia, 7.9% [25]. However, our finding was higher than studies reported from Thailand 6.5% [26] and India 5.5% [27]. In contrast, the finding was lower than studies conducted in Nigeria 15.3% [28], Sudan, 25.5% [1], 15.1% Zambia [22], 30% Iran [29], 34.1% Japan [30], and 41.5% Aden Yemen [31]. This difference may be due to the geographical area, socioeconomic conditions, seasonal variations, indoor air pollution, vaccination status, study design, sample size, and sampling technique [11, 32].

In this study, all *S*. *pyogenes* isolates were sensitive to penicillin. This result was consistent with previous studies reported from different parts of Ethiopia [18–20] and studies from China [3], Iran [29], Israel [13], Zambia [22], and Thailand [26]. In addition, the current study revealed that *S*. *pyogenes* was 100% sensitive to amoxicillin, which was similar to a previous study in Jimma, Ethiopia, that reported *S*. *pyogenes* 100% sensitivity to amoxicillin [19]. In contrast, a study conducted in Sudan reported that 58.8% of the isolates were resistant to amoxicillin [1]. Majority of the *S*. *pyogenes* isolates in the current study were also susceptible to the actions of azithromycin (86.4%), cefepime (90.9%), and clindamycin (95.5%). This result was higher than those study's results reported from Bahir Dar, Northern Ethiopia [18], and Gondar, Northwest Ethiopia [20].

The relatively higher level of resistance in the study from Bahir Dar may be due to a difference in the level of antibiotic use in Bahir Dar [18] and Gondar [20] studies.

In the contrast, the isolates demonstrated resistance of 36.4%, 31.8%, and 27.3% to tetracycline, ceftriaxone, and erythromycin, respectively. Similarly, *S*. *pyogenes* was found to be resistant to those antibiotics in other studies. The current tetracycline finding was greater than studies conducted in Gondar, Northwest Ethiopia, 21.7% [20], and Bahir Dar, Northern Ethiopia, 14.3% [18], but lower than Jimma, Southwest Ethiopia, 52.5% [19]. Additionally, the rate of erythromycin resistance is higher than in previous studies conducted in Ethiopia, Jimma [19], no resistance, Gondar 4.3% [20], and Bahir Dar 21.4% [18], and other countries like India with no resistance [27], Thailand 18.2% [26], but less than the finding from Iran 37.2% [29]. Similarly, the finding of ceftriaxone resistance was greater than a study conducted in Gondor, Ethiopia, 13% [18], but lower than a study in Thailand with no resistance [27]. Furthermore, 22.7% of the *S*. *pyogenes* isolates in this study were found to be multidrug resistant (MDR), which was marginally higher than the 21.3% found in a previous study conducted in Bahir Dar, Northern Ethiopia [18]. The observed variations in antibiotic sensitivity and phenotypic multidrug resistance in *S*. *pyogenes* could potentially be attributed to noncompliance with medication regimens or excessive drug consumption within the study area, as well as empirical pharyngitis treatment practices [33]. Furthermore, the primary emergence of MDR secondary to drug resistance is maybe due to beta-lactamase enzyme production, which carries genes specific to drug resistance, modifies antimicrobial targets, and activates the drug efflux pump of *S*. *pyogenes* [2, 12].

According to the associated factors, in the current study, boys were 3.91 times more likely than females to experience *S*. *pyogenes* acute pharyngitis (*P* value = 0.015). This result is in line with other studies reported from Taiwan [34] and Japan [30] which revealed that *S*. *pyogenes* pharyngitis is more associated with boys than girls. Similarly, the review studies conducted in China [35, 36] and United States [37, 38] also stated that *S*. *pyogenes* infection was more prevalent in boys than in girls. However, this result was at odds with previous Ethiopian studies carried out in Jimma [19], Gojam [20], and Bahir Dar [18] which, despite being statistically not significant, reported a higher prevalence of *S*. *pyogenes* pharyngitis in boys than in girls. This difference may be due to variations in study subjects, living standards, parental occupation, seasonal variations, and immune status between males and females [11, 39]. Concerning clinical factors, the presence of palatal petechiae and tonsillar swelling or exudate was significantly associated to children with *S*. *pyogenes* acute pharyngitis with *P* values of 0.037 and 0.007, respectively. This finding was in agreement with the previous studies of Jimma, Southwest Ethiopia [19], and Gondar, Northwest Ethiopia [20], and the other study from the United States of America [40]. However, our finding was in contradiction with the study from California [41] that stated a patient with no palatal petechiae clinical symptom was positive for *S*. *pyogenes*. In fact, palate petechiae and swollen tonsils or exudate were the most prevalent symptoms of *S*. *pyogenes* pharyngitis with sudden onset of fever and sore throat [29, 42, 43]. However, the geographical location and study subjects have an impact on the clinical characteristics of *S*. *pyogenes* pharyngitis [32].

### 4.1. Limitations of the Study

We acknowledge that there are some limitations in the study. The limitation of this study is its small size and small number of study sites which may underestimate the prevalence of *S*. *pyogenes*. Moreover, we did not perform the minimum inhibitory concentration (MIC) antimicrobial susceptibility test method rather than disc diffusion.

## 5. Conclusion

The prevalence of *S*. *pyogenes*, 10.3% in this study, was low compared to other studies. Penicillin and amoxicillin are effective drugs for the treatment of pediatric acute pharyngitis caused by *S*. *pyogenes*. Clindamycin and cefepime prescriptions are more suggested for case penicillin allergies materialize. Nevertheless, a significant rate of *S*. *pyogenes* resistance was noted to erythromycin, ceftriaxone, and tetracycline, which were the routine treatments for pediatric acute pharyngitis. Being boys is more susceptible to *S*. *pyogenes* acute pharyngitis than being girls. Palatal petechiae and tonsillar swelling or exudate are the clinical predictors for *S*. *pyogenes* acute pharyngitis. Therefore, the treatment of *S*. *pyogenes* acute pharyngitis among children should be depending on antimicrobial susceptibility patterns. Moreover, continued surveillance of *S*. *pyogenes* prevalence, antibiotic resistance, and risk factors is very important in controlling *S*. *pyogenes* pediatric acute pharyngitis. Developing specific guidelines for *S*. *pyogenes* and strengthening of laboratory infrastructure are significant to minimize the empirical treatment of *S*. *pyogenes* pediatric acute pharyngitis in Ethiopia.

## Figures and Tables

**Table 1:** Prevalence of *S*. *pyogenes* with respect to sociodemographic and clinical characteristics of pediatric patients with acute pharyngitis at HUCSH and Yirgalem General Hospital, 2022.

Sociodemographic variables	Categories	Culture result for *S*. *pyogenes* (n/213)		Frequency	(%)
		Positive *n* (%)	Negative *n* (%)		
Sex	Male	17 (8.0)	97 (45.5)	114	53.5
	Female	5 (2.3)	94 (44.1)	99	46.5

Age	<5 years old	12 (5.6)	83 (39.0)	95	44.6
	5–9 years old	9 (4.2)	83 (39.0)	92	43.2
	≥10 years old	1 (0.5)	25 (11.7)	26	12.2

Residence	Rural	0 (0)	14 (6.6)	14	6.6
	Urban	22 (10.3)	177 (83.1)	199	93.4

Children education level	Preschool	9 (4.2)	77 (36.2)	86	40.4
	Kindergarten	9 (4.2)	72 (33.8)	81	38.0
	Grade 1–4	4 (1.9)	42 (19.7)	46	21.6

Children living status	Mother and father	21 (9.9)	186 (87.3)	207	97.2
	Mother only	1 (0.5)	5 (2.3)	6	2.8

Family size	<5	12 (5.6)	124 (58.2)	136	63.8
	≥5	10 (4.7)	67 (31.5)	77	36.2

Parent educational level	No formal education	2 (0.9)	15 (7.0)	17	8.0
	Elementary	2 (0.9)	36 (16.9)	38	17.8
	High school	8 (3.8)	65 (30.5)	73	34.3
	Diploma and above	10 (4.7)	75 (35.2)	85	39.9

Parent occupational status	Government	2 (0.9)	39 (18.3)	41	19.2
	Private	15 (7.0)	123 (57.7)	138	64.8
	Farmer	1 (0.5)	10 (5.2)	11	5.2
	House worker	4 (1.9)	19 (8.9)	23	10.8

Non separated living room and kitchen	Yes	5 (2.3)	41 (19.2)	46	21.6
	No	17 (8.0)	150 (70.4)	167	78.4

Number of windows in the house	1	2 (0.9)	36 (16.9)	38	17.8
	2	13 (6.1)	82 (38.5)	95	44.6
	≥3	7 (3.3)	73 (34.3)	80	37.6

*Clinical variables*					
Vaccine complete	Yes	19 (8.9)	162 (76.1)	181	85.0
	No	3 (1.4)	29 (13.6)	32	15.0

Previous history of tonsillitis	Yes	15 (7.0)	122 (57.3)	138	64.8
	No	7 (3.3)	69 (32.4)	75	35.1

Previous otitis media	Yes	3 (1.4)	27 (12.7)	17	8.0
	No	19 (8.9)	164 (77.0)	196	92.0

Previous conjunctivitis	Yes	3 (1.4)	22 (10.3)	25	11.7
	No	19 (8.9)	169 (79.3)	188	88.3

Enlarged anterior cervical lymph node	Yes	8 (3.8)	75 (35.2)	83	39.0
	No	14 (6.6)	116 (54.5)	130	61.0

Palatal petechial	Yes	10 (4.7)	128 (60.1)	138	64.8
	No	12 (5.6)	63 (29.6)	75	35.2

Tonsillar swelling or exudate	Yes	14 (6.6)	76 (35.7)	90	42.3
	No	8 (3.8)	115 (54.0)	123	57.7

Cough	Yes	14 (6.6)	100 (46.9)	114	53.6
	No	8 (3.8)	91 (42.7)	99	46.5

Fever	>3 days	13 (6.1)	92 (43.2)	105	49.3
	≤3 days	9 (4.2)	99 (46.5)	108	50.7

Headache	Yes	6 (2.8)	48 (22.5)	54	25.4
	No	16 (7.5)	143 (67.1)	159	74.6

Nasal flow	Yes	15 (7.0)	91 (42.7)	106	49.8
	No	7 (3.3)	100 (46.9)	107	50.2

Abdominal pain	Yes	4 (1.9)	51 (23.9)	55	25.8
	No	18 (8.5)	140 (65.7)	158	74.2

Vomiting	Yes	11 (5.2)	128 (60.1)	139	65.3
	No	11 (5.2)	63 (29.6)	74	34.7

**Table 2:** Multiple drug resistant of *S*. *pyogenes* isolated from pediatric with acute pharyngitis at HUCSH and Yirgalem General Hospital, 2022.

Resistance of antibiotics	*N* (%)	MDR (*n* (%))
Ceftriaxone	3 (13.6)	
Tetracycline	3 (13.6)	
Erythromycin	2 (9.1)	
Ceftriaxone, erythromycin, tetracycline	1 (4.5)	1 (4.5)
Clindamycin, azithromycin, tetracycline	1 (4.5)	2 (9.1)
Erythromycin, ceftriaxone, cefepime, tetracycline,	1 (4.5)	
Ceftriaxone, chloramphenicol, erythromycin, tetracycline	1 (4.5)	2 (9.1)
Erythromycin, ceftriaxone, cefepime, tetracycline	1 (4.5)	

Key: MDR-Multidrug resistant.

**Table 3:** Bivariate and multivariable logistic regression analysis of factors associated with *S*. *pyogenes* acute pharyngitis at HUCSH and Yirgalem General Hospital, 2022.

Socio-demographic characteristics	Categories	Culture result for *S*. *pyogenes* (*n*/213)		COR (95% CI)	*P* value	AOR (95% CI)	*P* value
		Positive *n* (%)	Negative *n* (%)				
Sex	Male	17 (8.0)	97 (45.5)	3.29 (1.168–9.291)	0.024	3.91 (1.297–11.792)	0.015^∗^
	Female	5 (2.3)	94 (44.1)	1		1	

Age	<5 years	12 (5.6)	83 (39.0)	0.28 (0.034–2.233)	0.228	0.23 (0.027–1.972)	0.181
	5–9 years	9 (4.2)	83 (39.0)	0.37 (0.045–3.054)	0.355	0.25 (0.028–2.230)	0.215
	≥10 years	1 (0.5)	25 (11.7)	1			

Residence	Rural	0 (0)	14 (6.6)	—	—		
	Urban	22 (10.3)	177 (83.1)	—	—	—	—

Children education level	Preschool	9 (4.2)	77 (36.2)	0.82 (0.237–2.805)	0.745	—	—
	Kindergarten	9 (4.2)	72 (33.8)	0.76 (0.221–2.627)	0.667	—	—
	Grade 1–4	4 (1.9)	42 (19.7)	1			

Children living status	Mother and father	21 (9.9)	186 (87.3)	1.77 (0.197–15.890)	0.609	—	—
	Mother only	1 (0.5)	5 (2.3)	1		—	—

Family size	<5	12 (5.6)	124 (58.2)	0.65 (0.266–1.579)	0.337	—	—
	≥5	10 (4.7)	67 (31.5)	1		—	—

Parent educational level	No formal education	2 (0.9)	15 (7.0)	1			
	Elementary	2 (0.9)	36 (16.9)	2.40 (0.309–18.651)	0.403	—	—
	High school	8 (3.8)	65 (30.5)	1.08 (0.208–5.630)	0.924	—	—
	Diploma and above	10 (4.7)	75 (35.2)	1.00 (0.199–5.034)	1.000	—	—

Parent occupational status	Government	2 (0.9)	39 (18.3)	1			
	Private	15 (7.0)	123 (57.7)	4.11 (0.690–24.435)	0.121	0.38 (0.079–1.808)	0.223
	Farmer	1 (0.5)	10 (5.2)	1.73 (0.518–5.755)	0.374	0.26 (0.015–4.587)	0.359
	House worker	4 (1.9)	19 (8.9)	2.11 (0.207–21.449)	0.530	0.21 (0.034–1.360)	0.102

Nonseparated living room and kitchen	Yes	5 (2.3)	41 (19.2)	1			
	No	17 (8.0)	150 (70.4)	1.08 (0.375–3.091)	0.892	—	—

Number of windows in the house	1	2 (0.9)	36 (16.9)	1		1	
	2	13 (6.1)	82 (38.5)	0.35 (0.075–1.634)	0.182	0.21 (0.034–1.303)	0.094
	≥3	7 (3.3)	73 (34.3)	0.58 (0.114–2.932)	0.509	0.42 (0.064–2.804)	0.372

Key: AOR: adjusted odds ratio, COR: crude odds ratio, CI: confidence interval, *Note.*^∗^Indicates the significant relation between variables, *P* value <0.05.

**Table 4:** Bivariate and multivariable logistic regression analysis of the clinical factor with *S*. *pyogenes* acute pharyngitis at HUCSH and Yirgalem General Hospital, 2022.

Clinical factors	Categories	Culture result for *S*. *pyogenes* (n/213)		COR (95% CI)	*P* value	AOR (95% CI)	*P* value
		Positive *n* (%)	Negative *n* (%)				
Vaccination complete	Yes	19 (8.9)	162 (76.1)	0.88 (0.245–3.173)	0.848	—	—
	No	3 (1.4)	29 (13.6)	1			

Previous history of tonsil	Yes	15 (7.0)	122 (57.3)	0.82 (0.321–2.122)	0.690	—	—
	No	7 (3.3)	69 (32.4)	1			

Previous otitis media	Yes	3 (1.4)	27 (12.7)	1.00		—	—
	No	19 (8.9)	164 (77.0)	0.95 (0.266–3.463)	0.949	—	—

Previous conjunctivitis	Yes	3 (1.4)	22 (10.3)	1			
	No	19 (8.9)	169 (79.3)	1.21 (0.332–4.433)	0.770	—	—

Enlarged anterior cervical lymph node	Yes	8 (3.8)	75 (35.2)	1		—	—
	No	14 (6.6)	116 (54.5)	0.88 (0.354–2.209)	0.791	—	—

Palatal petechiae	Yes	10 (4.7)	128 (60.1)	1		1	
	No	12 (5.6)	63 (29.6)	0.41 (0.168–1.001)	0.045^∗^	0.35 (0.131–0.938)	0.037^∗^

Tonsillar swelling or exudate	Yes	14 (6.6)	76 (35.7)	2.65 (1.060–6.616)	0.032^∗^	3.83 (1.446–10.188)	0.007^∗^
	No	8 (3.8)	115 (54.0)	1			

Cough	Yes	14 (6.6)	100 (46.9)	1.59 (0.639–3.972)	0.315	—	—
	No	8 (3.8)	91 (42.7)	1		—	—

Fever	>3 days	13 (6.1)	92 (43.2)	1.55 (0.634–3.808)	0.332	—	—
	≤3 days	9 (4.2)	99 (46.5)	1		—	—

Headache	Yes	6 (2.8)	48 (22.5)	1		—	—
	No	16 (7.5)	143 (67.1)	1.12 (0.414–3.017)	0.827	—	—

Nasal flow	Yes	15 (7.0)	91 (42.7)	2.36 (0.919–6.034)	0.068^∗^	0.41 (0.145–1.129)	0.084
	No	7 (3.3)	100 (46.9)	1		1	

Abdominal pain	Yes	4 (1.9)	51 (23.9)	1		—	—
	No	18 (8.5)	140 (65.7)	0.61 (0.197–1.888)	0.387	—	—

Vomiting	Yes	11 (5.2)	128 (60.1)	0.49 (0.202–1.197)	0.112^∗^	1.43 (0.513–3.995)	0.493
	No	11 (5.2)	63 (29.6)	1		1	

OR: odds ratio, CI: confidence interval. *Note*. ^∗^Indicates the significant relation between variables, *P* value <0.05.

## Data Availability

The datasets used and/or analyzed during the current study are available from the corresponding author on reasonable request.

## Abbreviations

HUCSH: Hawassa University comprehensive specialized hospital
IRB: Institutional review board
SNNPR: Southern nations, nationalities, and peoples region.
